# The participation of NMDA receptors, PKC, and MAPK in the formation of memory following operant conditioning in *Lymnaea*

**DOI:** 10.1186/1756-6606-3-24

**Published:** 2010-08-31

**Authors:** David Rosenegger, Ken Lukowiak

**Affiliations:** 1 Department of Physiology and Biophysics, Hotchkiss Brain Institute, University of Calgary, 3330 Hospital Drive NW, Calgary, Alberta, T2N 4N1, Canada

## Abstract

**Background:**

Memory is the ability to store, retain, and later retrieve information that has been learned. Intermediate term memory (ITM) that persists for up to 3 h requires new protein synthesis. Long term memory (LTM) that persists for at least 24 h requires: DNA transcription, RNA translation, and the trafficking of newly synthesized proteins. It has been shown in a number of different model systems that NMDA receptors, protein kinase C (PKC) and mitogen activated protein kinase (MAPK) are all involved in the memory formation process.

**Results:**

Here we show that snails trained in control conditions are capable of forming, depending on the training procedure used, either ITM or LTM. However, blockage of NMDA receptors (MK 801), inhibition of PKC (GF109203X hydrochloride) and MAPK activity (UO126) prevent the formation of both ITM and LTM.

**Conclusions:**

The injection of either U0126 or GF109203X, which inhibit MAPK and PKC activity respectively, 1 hour prior to training results in the inhibition of both ITM and LTM formation. We further found that NMDA receptor activity was necessary in order for both ITM and LTM formation.

## Background

The formation of memories following learning is hypothesized to be dependent on both the altered strength of synaptic connections between neurons and changes to intrinsic membrane properties of those neurons that are necessary for memory formation. For memories lasting longer that a few minutes, the changes in synaptic strength and neuron excitability require a physical alteration of both the synaptic and membrane complement of proteins. Strong evidence for this exists in the form of numerous studies showing the requirement of new protein synthesis and altered gene activity in order for long-term memories (LTM) to form [1-4]. Additionally, much work has gone into identifying the various signaling cascades that ultimately lead to the production of new proteins and thus memory [5]. Among the numerous proteins identified to be important in memory formation are a subset of molecules (e.g. NMDA receptors, PKC, MAPK) that have been shown to play key roles across a number of species.

The fresh water snail *Lymnaea stagnalis *has been used as a model system to investigate the mechanisms underlying LTM formation. Predominantly, these studies have focused on the either classical conditioning of feeding behaviours or operant conditioning of aerial respiratory behaviour [6-9]. Owing to its relatively simple nervous system consisting of large identifiable neurons, a detailed description of the neuronal circuitry underlying these behaviours has been elucidated. Thus, we have a good understanding of the electrophysiological correlates of LTM formation in *Lymnaea *[10-12]. However, considerably less is known about the molecular mechanisms underlying memory formation in this animal.

*Lymnaea *is a bimodal breather; that is, it is able to satisfy its respiratory requirements both cutaneously and aerially. Aerial respiration is accomplished at the water-air interface via the snail opening its respiratory orifice, the pneumostome, while at the same time contracting and relaxing its respiratory muscles [13]. Snails typically only resort to increased aerial respiration when their environment becomes hypoxic [13]. Aerial respiratory behavior, as a result, can there-fore be operantly conditioned in a hypoxic environment. Conditioning results in fewer attempted openings in memory tests and serves as our operational definition of memory. Since snails can still perform cutaneous respiration in hypoxia, snails trained not to perform aerial respiration are not harmed as a result of training [13-15]. Depending on the training procedure used in *Lymnaea*, either intermediate term memory (ITM; persisting up to 3 h) or LTM (persisting at least 24 h) results following operant conditioning of aerial respiratory behavior [14-18]. We have also found that while both ITM and LTM depend upon new protein synthesis there is an additional requirement of altered gene activity (i.e. transcription) for LTM formation [19-23]. Importantly, we have also shown the necessary requirement for the soma (i.e. the genes) of right pedal dorsal 1 (RPeD1), the neuron which is responsible for initiating rhythmogenesis of the aerial respiratory central pattern generator, to be present in order for LTM to form [24].

Included among the molecules which have been found to be required for memory formation across several memory types, and numerous species are the N-methyl-D-aspartate (NMDA) receptors [25-28], protein kinase C (PKC) members [29-31], and the mitogen activated protein kinase (MAPK) family [32-35]. Previously, we had shown that bryostatin, a PKC agonist, could enhance LTM formation following a single 0.5 h training session, which typically only results in ITM in *Lymnaea *[36]. Even more recently we have shown that an operant conditioning paradigm of the aerial respiratory behaviour that leads to LTM formation causes a significant increase in the expression of mitogen activated protein kinase kinase 1 (MEKK1), a member of the MAPK family of proteins, and the novel expression of the epsilon isoform of PKC [37]. NMDA receptors have long been studied for their role in the processes of synaptic plasticity and memory formation. In Lymnaea this avenue of research is in its infancy, with the recent cloning of NMDA receptors [38], and first experiment showing NMDAr to be required for LTM formation in a classical conditioning paradigm [39].

Previously our laboratory [40] used a drug often associated with the NMDA receptor, ketamine; and found that ketamine administration either just before or up to 2 h a after a training event (a 1-trial training procedure) blocked the formation of LTM but not ITM. Since the concentration of ketamine used in that study (a concentration of 0.004 mg/ml ketamine when bath applied in hypoxic-pond water) did not interfere with aerial respiratory behaviour or ITM formation, but only LTM formation it was concluded that ketamine's effect on LTM formation was due to its acting at the required gene transcription processes in neurons (e.g. RPeD1) necessary for LTM formation. Thus, NMDA receptor activity in that series of experiments was thought not to be involved in the formation of memory. In this present series of studies, however, we set out to test the requirement of an NMDA receptor, as well as whether intracellular cascades involving either PKC, and MAPK activity in *Lymnaea *are required for the formation of memory as a result of operant conditioning of the aerial respiratory behaviour.

We show here that the activation of the NMDA receptor as well as the subsequent intracellular cascades involving PKC and MAPK are required in order to allow LTM formation following operant conditioning of aerial respiration in *Lymnaea*. With this we hope to develop a more complete picture of the memory formation process in *Lymnaea *from the behavioural to the molecular level.

## Methods

### Animals

The fresh water pond snail *Lymnaea stagnalis *was used in the experiments reported here. Animals were bred and maintained at the facilities in the University of Calgary, from a colony initially set up at the Vrije University in The Netherlands from snails collected in the wild in the 1950's. Adult animals with a shell size larger than 20 mm were used in all experiments. Animals were maintained, and all experiments were performed at room temperature (~20-21°C).

### Training Protocols

Animals were trained as previously described [9,36]. Briefly, individually labeled snails were placed into a 1L beaker containing 500 mL of water made hypoxic by bubbling N_2 _through it for at least 20 min prior to training (N_2 _is also bubbled through the water through out the training and memory test sessions). Animals are allowed to acclimatize for 10 min prior to training. Operant conditioning is accomplished by applying a tactile stimulus to the pneumostome area each time aerial respiration is attempted. With training animals learn to decrease the number of pneumostome openings. Memory is defined to be present if the number of attempted pneumostome openings in the 'test' session is significantly less than the number of attempted openings in the initial training session [13]. This decrease in attempted pneumostome openings is dependent on the contingency of the tactile stimulus to pneumostome opening, as tactile stimulus alone (i.e. Yoked controls snails) does not result in a decrease of pneumostome openings [13]. Two different training protocols were used, a single 30 min training session, and a single 60 min training session. The 30 min training session only results in a memory persisting for ~3 h. This has been defined as intermediate term memory (ITM). ITM is dependent on new protein translation [19]. The 60 min training session produces both ITM and long-term memory (LTM) that lasts for > 24 hours. LTM is dependent on both altered gene activity and new protein synthesis [19].

### Breathing Observations

Breathing observations were performed to ensure that each drug treatment did not significantly affect the baseline aerial respiratory behaviour of the animals. This ensures that any changes observed to aerial respiration are not simply the result of negative drug interactions. Breathing observations were performed by placing labeled animals into a beaker of hypoxic pond water, and then recording the total time each animal spent performing aerial respiration. These observations were done both before and a after drug treatment, and then the average of breathing times for each session is compared to determine if any significant changes occurred.

### Drug treatments

Drugs were administered by injection into the hemocoele via the foot of the snails. It was assumed that animals of the same size have a similar hemolymph volume, and thus the drugs will be at a similar final concentration in the animals. In this study we used the non-competitive N-methyl D-aspartate (NMDA) receptor antagonist MK801 (Sigma) (0.1 mL of 150 μM MK-801 dissolved in saline), the protein kinase C (PKC) inhibitor GF109203X hydrochloride (Sigma) (0.1 mL of 0.4 μM GF109203X dissolved in saline), and the mitogen-activated protein kinase kinase (MAPKK or MEK) inhibitor U0126 (Promega) (0.2 mL of 500 μM U0126 dissolved in saline and 20% methanol; i.e. the vehicle), which inhibits the activation of MAPK (also called ERK1/2) [41]. Drugs were administered at 1 hour prior to training to determine their effects on the memory formation process.

### Statistics

The experimental data in this paper were analyzed using standard accepted statistical methods. Breathing observation data were analyzed using a repeated measures 1-way ANOVA and *post-hoc *Tukey's multiple comparisons test. All other data were analyzed by the use of a paired t-test. For all analysis data were considered significant if p < 0.05.

## Results

### Saline and vehicle injected control snails

**Figure 1:**
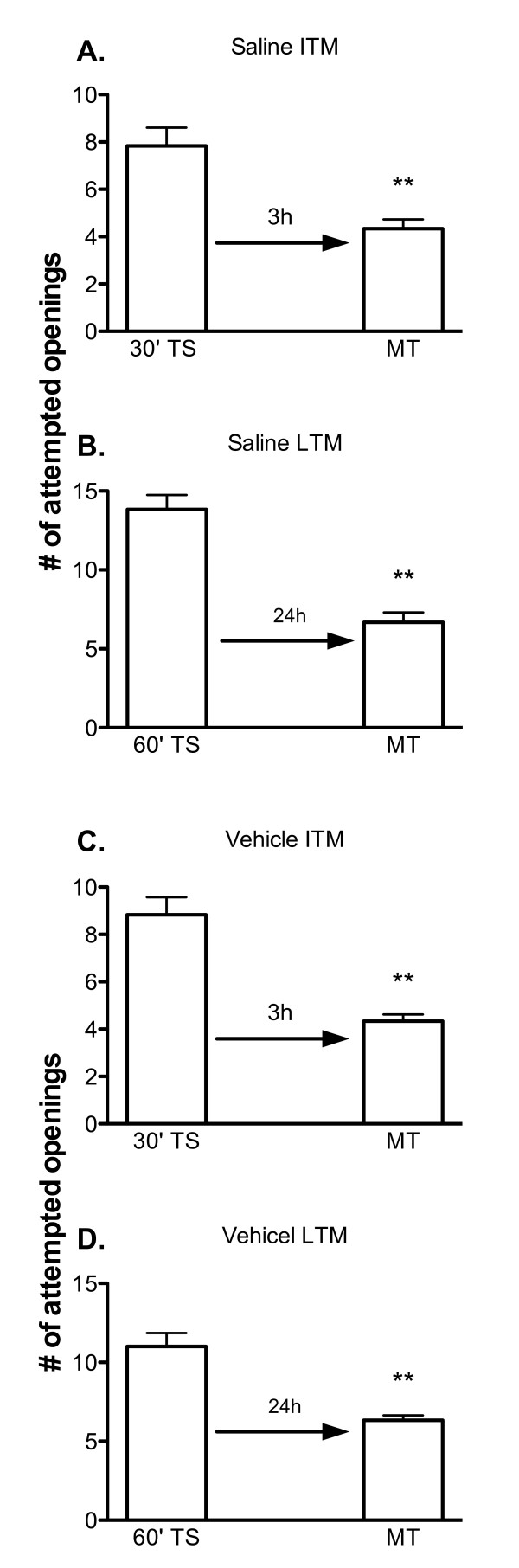
**Saline and vehicle injected snails exhibit ITM and LTM**. A) Snails injected with saline 1 h prior to a 30 min training session (TS) exhibit intermediate-term memory (ITM) when given a memory test (MT) at 3 h (30' TS vs. 3 h MT, p < 0.01, n = 12). B) Snails injected with saline 1 h prior to a 60 min training session (TS) exhibit long-term memory (LTM) when tested for memory (MT) 24 h later. LTM formation was not inhibited by saline injection (60' TS vs. 24 h MT p < 0.001, n = 12). C) Vehicle injection had no effect on the ability to form ITM (30'TS vs. 3 h MT p < 0.001, n = 12). D) LTM formation was not inhibited by vehicle injection (60' TS vs. 24 h MT p < 0.001, n = 12). ** p < 0.01.

Stress alters memory formation in *Lymnaea *[18]; thus, we had to first demonstrate that injection of snails with either saline, which we use to dissolve MK 801 and GF109203X, or vehicle (saline and 20% methanol) which we use to dissolve UO126 would not block either ITM or LTM formation. As can be seen in Figure 1 both saline injected and vehicle injected snails have the capacity to form both ITM and LTM. A 30 min training session results in a memory that persists for 3 h (i.e. ITM); whilst a 60 min training session results in a memory that persists for 24 h (i.e. LTM). Thus, injecting snails 1 h before training did not cause sufficient stress to alter the snails ability to form memory. In addition, vehicle injection also did not alter the ability of the snails to form memory.

#### NMDA

**Figure 2:**
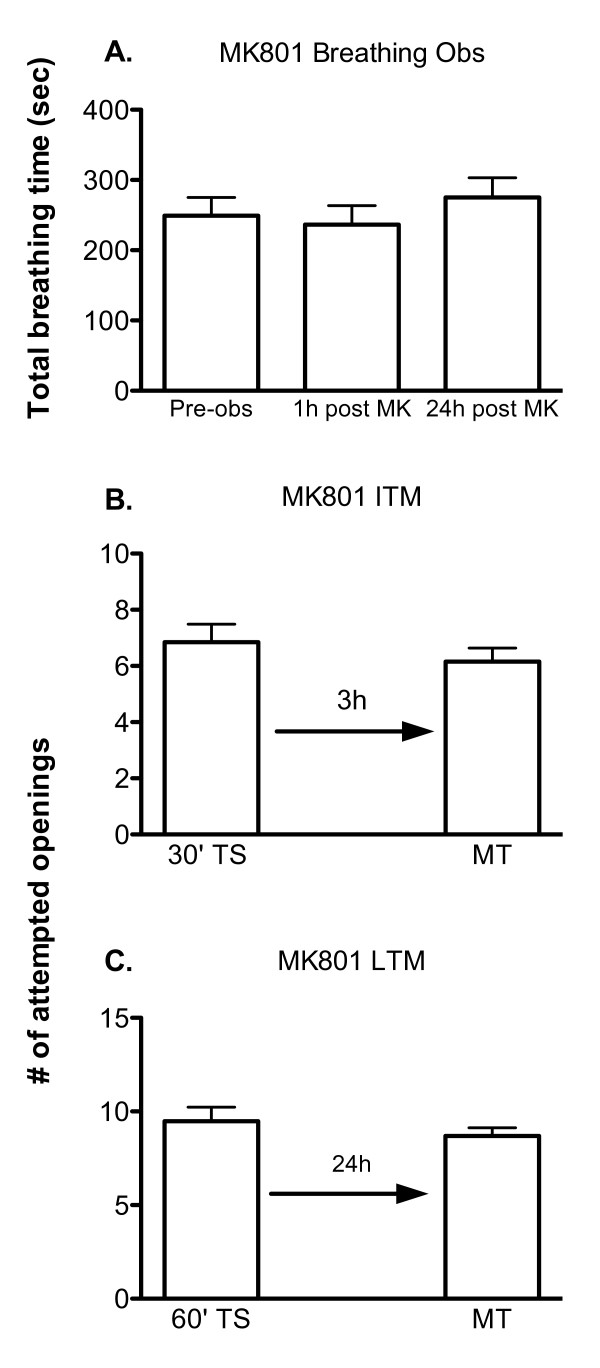
**MK-801 injected snails do not exhibit ITM or LTM**. A) MK-801 injection does not alter aerial respiratory behaviour. Snails were placed in hypoxic pond water and the mean (± SEM) total breathing time was calculated before (pre-obs) and 1 h (1 h post MK) and 24 h (24 h post MK) a after they were injected with MK-801 (0.1 mL of 150 μM). The injection of MK-801 1 h before the breathing observation session did not alter aerial respiratory behaviour (ANOVA_2,17 _F = 0.8922, p > 0.05). B) MK-801 inhibits the formation of ITM when administered 1 hour prior to training (30' TS vs. 3 h MT, p = 0.2856, n = 20). C) LTM formation is also blocked by injecting animals with MK-801 prior to training (60' TS vs. 24 h MT p = 0.3555, n = 23).

As a first step in determining the effect of MK-801 on memory formation, we first performed breathing observations to determine if this drug significantly altered baseline breathing behaviour. This was an important control to perform, as memory is experimentally defined to be present if there is a significant reduction in the number of attempted pneumostome openings, and thus if a drug significantly reduces breathing it could interfere with the interpretation of the results. A naïve cohort of animals (n = 18) were subjected to three 30 min breathing observations in hypoxic pond water (Figure 2A). The first observation was performed prior to any treatment to gain a baseline of the normal rate of aerial respiration under hypoxic conditions. Next animals were injected with 0.1 mL of 150 μM MK-801, followed by two more breathing observations performed 1 hour and 24 hours a after MK-801 injection. As shown in Figure 2A this concentration of MK-801 did not significantly alter the aerial respiratory behaviour of the animals (ANOVA_2,17 _F = 0.8922, p > 0.05).

Given that this drug concentration did not alter baseline aerial respiratory behaviour, we next tested whether it affected the memory formation process(es). As described above two different training regimes were used. A single 30 min training session produces ITM but not LTM. ITM in *Lymnaea *is dependent on new protein translation and not altered gene activity. On the other hand the single 60 min training session produces an LTM that requires both altered gene activity and the translation of new proteins (19).

We first wished to determined if ITM memory formation in *Lymnaea *required NMDA receptor activity (Figure 2B). A cohort of naive animals (n = 20) was injected with 0.1 mL of 150 μM MK-801 dissolved in saline. One hour a after injection they were given a single 30 min training session and then were tested for ITM 3 hours later. When tested the MK-801 injected animals did not show any significant decrease in the number of attempted pneumostome openings, and thus had no memory (30' TS vs. 3 hr MT, p = 0.2856). Saline injected animals (n = 12: Fig 1) that received the same training paradigm, on the other hand, significantly reduced the number of attempted pneumostome openings during the test session and were thus formed ITM (30' TS vs. 3 hr MT, p < 0.01). These data indicate that NMDA receptor activity in *Lymnaea *is necessary for the formation of ITM following the 30 min operant conditioning training session.

We next tested if the same was true for LTM formation (Figure 2C). Animals received injections of either MK-801 (n = 23), or saline one hour prior to the single 60 min operant conditioning training session. When memory was tested 24 hours a after the operant conditioning training we found that the MK-801 injected animals showed no significant change in the number of attempted pneumostome openings (60' TS vs. 24 hr MT p = 0.3555), while the saline injected animals (Figure 1) showed a significant reduction (60' TS vs. 24 hr MT p < 0.001). Thus, MK-801 injected animals failed to form LTM while saline injected animals formed LTM. These data indicate a requirement for NMDA receptor activity for the formation of LTM following operant conditioning of aerial respiration.

#### PKC

**Figure 3:**
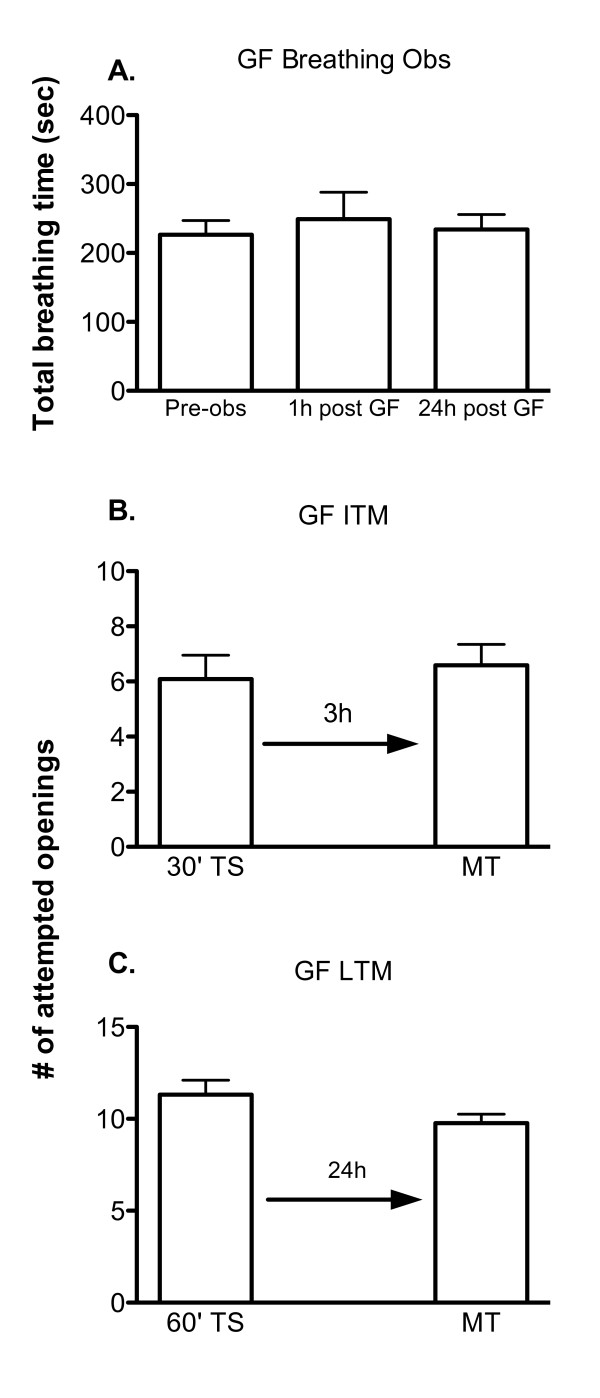
**Inhibiting PKC activity prevents ITM and LTM formation**. A) Basal aerial respiratory behaviour is not significantly altered at 1 (1 h post GF) or 24 (24 h post GF) hours a after injection of the PKC inhibitor GF109203X (0.1 mL of 0.4 μM). B) Injection of GF109203X an hour prior to training (TS) effectively inhibited the formation of ITM (30' TS vs. 3 h MT p = 0.6203, n = 12). C) LTM formation is also abolished by GF109203X injection prior to training (TS) (60' TS vs. 24 h MT p = 0.1181, n = 22).

Having shown the requirement of NMDA receptor activity in the formation of both ITM and LTM, we next sought to determine whether PKC activity is required for the formation of either ITM or LTM following operant conditioning of aerial respiratory behaviour in *Lymnaea*. These experiments (Figure 3) followed the same set of experimental protocols as the MK-801 experiments. That is, breathing observations were first performed (Figure 3A) to determine the concentration of the drug to be used. Thus, a 30 min observation session was done to determine baseline aerial respiratory behaviour. Next animals (n = 10) were injected with 0.1 mL of 0.4 μM GF109203X, and 30 minute breathing observation sessions were performed again at 1 h and 24 h a after injection of the drug. There was no significant difference in the mean total breathing time between the three observation sessions (ANOVA_2,9 _F = 0.2939, p > 0.05), indicating that this concentration of drug did not alter breathing behaviour.

Next we injected another naïve cohort of animals (n = 12) with the same concentration of GF109203X followed by a 30 min operant conditioning training session (Figure 3B). We then tested for ITM 3 h later (3 h MT) and found that there was no significant difference in the number of attempted pneumostome openings compared to the first training session (30'TS) (30' TS vs. 3 hr MT p = 0.6203). Saline injected control animals, however, formed ITM following a 30 minute training session. We thus concluded that this concentration of drug prevent ITM from being formed.

Having demonstrated that GF109203X prevented the formation of ITM we next wished to determine if GF109203X would also prevent the formation of LTM. Thus, a new cohort of naive snails (n = 22) was first injected with GF109203X and then 1 h later subjected to a 1 h operant conditioning training session (60' TS). A memory test session (24 h MT) was then given to these snails 24 h later. As can be seen (Figure 3C) memory was not observed. That is, the number of attempted pneumostome openings in the memory test session was not statistically different (60' TS vs. 24 hr MT p = 0.1181) from the number of attempted openings in the initial 1 h training session. Saline injected control snails exhibited LTM when tested 24 h later. Thus we conclude that the injection of GF109203X prior to operant conditioning training blocks the formation of LTM.

#### MAPK

**Figure 4:**
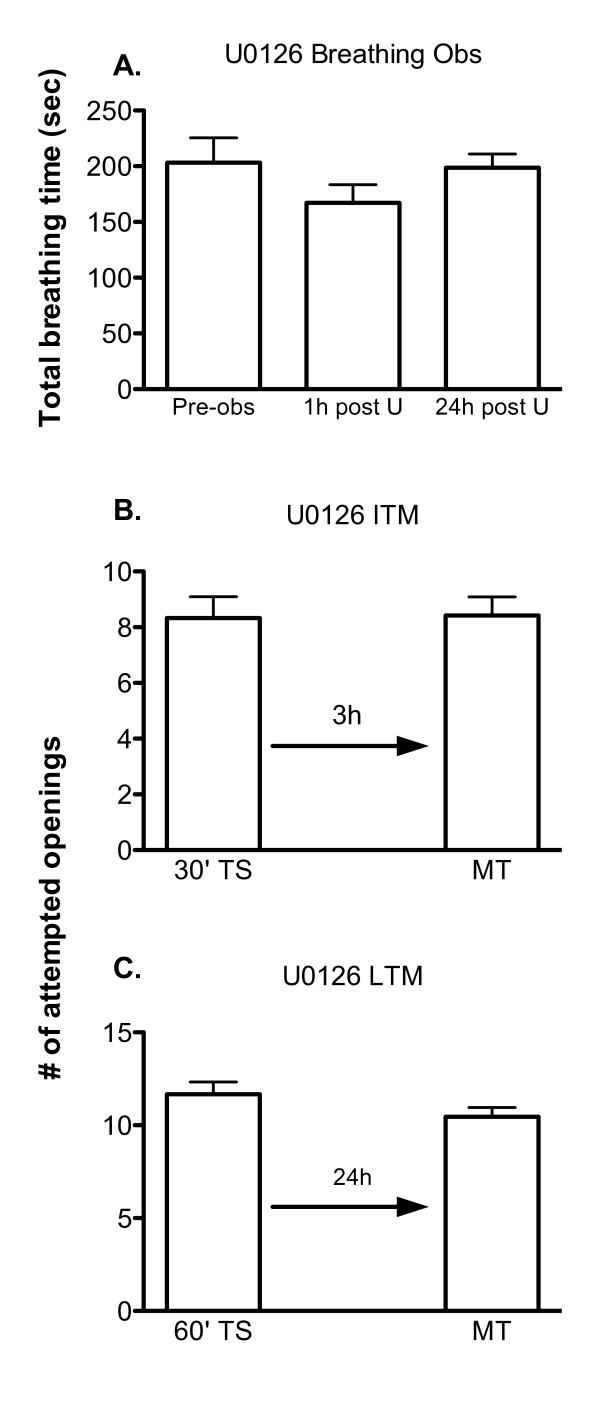
**Inhibition of MAPK activity prevents the formation of ITM and LTM**. A) U0126 treatment (0.2 ml injection of 500 μM) does not impair the ability to perform aerial respiration, as no significant difference in total breathing time was observed (ANOVA_2,9 _F = 0.1741, p > 0.05, n = 10). B) ITM formation is obstructed by injection of U0125 1 hour prior to training (TS) (30' TS vs. 3 h MT p = 0.9201, n = 12). C) LTM formation is also blocked by injecting animals with U0126 prior to training (TS) (60' TS vs. 24 h MT p = 0.1630, n = 24).

The next series of experiments (Figure 4) was designed to determined if MAPK activity was necessary for the formation of memory following operant conditioning in *Lymnaea*. To test if memory formation required the activation of MAPK we used the drug U0126 which works by inhibiting the activator of MAPK, MEK. Breathing observations were first performed as with the other two drugs (Figure 4A). A baseline breathing observation was made and then the naive animals (n = 10) were injected with 0.2 mL of 500 μM U0126 dissolved in saline and methanol, followed by breathing observation sessions 1 h and 24 h later. This concentration of drug did not alter aerial respiratory behaviour, as there was no significant change in the average total breathing time of the animals (ANOVA_2,9 _F = 0.1741, p > 0.05).

We then proceeded to determine whether UO126 blocked the formation of ITM and LTM (Figure 4B, C). Thus a naïve cohort of animals (n = 12) was injected with U0126 1 h prior to a 30 minute operant conditioning training session and memory was tested in these snails 3 h later (Figure 4B). Snails injected with vehicle (n = 12; Figure 1) exhibit ITM (30' TS vs. 3 hr MT p < 0.001). However, snails injected with UO126 failed to show ITM. That is, the number of attempted pneumostome openings in the 3 h memory test session (3 hr MT) was not statistically different from the number of attempted openings in the 30 min operant conditioning training session (30' TS vs. 3 hr MT p = 0.9201).

Having demonstrated that UO126 blocks the formation of ITM we were interested to determine if it would also block LTM formation. Therefore we injected U0126 into a new naive cohort of snails (n = 24); and then 1 h later these snails were trained using the 60 minute training procedure. As can be seen (Figure 4C) memory was not present. That is, the number of attempted pneumostome openings in the 24 h memory test (24 h MT) was not significantly different than the number of attempted openings in the 60 minute training session (60' TS vs. 24 hr MT p = 0.1630) Vehicle control animals (Figure 1; n = 12) were once again fully able to form LTM (60' TS vs. 24 hr MT p < 0.001). Thus we conclude that the injection of UO126 is sufficient to block formation of LTM.

## Discussion

In this study we show that an NMDA receptor blocker, and inhibitors of the intracellular cascades involving PKC, and MAPK respectively are able to block the formation of both ITM and LTM. Previous work has shown that following operant conditioning of aerial respiration that ITM requires the translation of new proteins from pre-existing RNA, while LTM requires both the transcription of new RNA, and their translation into new proteins [19]. Thus, the data obtained using these blocking agents suggest that each of these molecules (i.e. NMDA receptors, PKC, and MAPK) play an important role in either signaling, initiating or maintaining processes that result in the necessary altered gene activity and new protein synthesis processes which are necessary for the formation of the memories that persist longer than a few minutes.

Alterations in NMDA receptor activity have been shown to be necessary for the induction of several forms of learning, and correlated synaptic plasticity in a variety of different animals [28,42-45]. Typical NMDA receptors have a voltage dependent magnesium block, have fairly slow kinetics, are permeable to calcium, and have binding sites for cofactors. These traits make the NMDA receptor well suited to associative plasticity mechanisms, specifically by allowing coincident and contingent stimuli to cause calcium entry through the activated, depolarized NMDA receptor which can then activate a variety of cell signaling cascades. Molecular and biophysical studies of *Lymnaea *NMDA receptors (LyNR) have revealed them to be widely expressed in CNS neurons (> 80%) including RPeD1 [38]. This is important as regards LTM formation following operant conditioning of aerial respiration, as RPeD1 is a neuron that is a necessary site for the formation of LTM, memory reconsolidation, memory extinction and forgetting [20-24]. Although LyNR are similar to mammalian NMDA receptors in several aspects, evidence suggests that they may lack the magnesium blocking mechanism. Despite some differing characteristics, recent work has demonstrated that NMDA-receptor activity is critical for the formation of a LTM as a result of a classical conditioning paradigm [39]. Here we show that memory formation in *Lymnaea *as a result of operant conditioning absolutely requires NMDA activity, as blocking NMDA receptors with MK-801 abolishes the ability to form memory without affecting the aerial respiratory behaviour. Thus, in *Lymnaea *as in other animals NMDA receptors play a pivotal role in synaptic modulation but not basal synaptic activity.

We previously found that another drug, associated with NMDA channel activity, ketamine, also altered memory formation in *Lymnaea *[40]. Ketamine's ability to act as a non-competitive antagonist at NMDA receptor sites is typically what is thought to endow it with its dissociative properties and its ability to disrupt long-lasting (i.e. > than a few minutes) memory formation [46]. More recently it has been demonstrated that ketamine may exert some of its effects via less studied mechanisms, such as by altering gene transcription [47-49]. In the Browning and Lukowiak 2008 [40] study the application of ketamine did not block ITM formation. It only blocked the formation of LTM. Since the associative learning and the formation of ITM were not altered by ketamine the authors hypothesized that ketamine's effect on LTM formation was due not to its ability to modify current flow through the NMDA receptor activated channels but rather ketamine's ability to directly block altered gene activity. Additionally, the previous study using ketamine as a blocker [40] employed a different training procedure to produce ITM and LTM. They used a one-trial training procedure which does not employ touching the pneumostome as it attempts to open, but rather uses a KCl-bath as an aversive stimulus that is contingent on the snail opening its pneumostome just once in an hypoxic environment. Whether ketamine would produce similar effects as we have shown here with MK801 has not yet been determined.

The PKC family of serine/threonine kinases can exert their actions by phosphorylating a variety of cellular targets. Early work in *Aplysia*, demonstrated that the synaptic facilitation induced by serotonin (5-HT) application to sensory neurons resulted in the translocation of PKC [50]. Further evidence of the role of PKC in memory formation was found with the discovery of the *Drosophila *PKC deficient mutant turnip which showed a severe reduction in the learning ability of the animal [51]. Since these studies there have been several others performed showing the requirement of PKC activity in a number of species and learning paradigms. For example, the activation of PKC has also been demonstrated to occur during classical conditioning of *Hermissenda *[52]. Furthermore, enhancing PKC activation greatly improves the acquisition of learning, and the duration of memory in this system [53]. We show here that PKC is required for the formation of both ITM and LTM following an associative learning training procedure that in control snails results in both ITM and LTM. We had hypothesized that PKC would be required for long-lasting memory formation following operant conditioning based in part on earlier studies performed in our laboratory. We had previously found that the duration of LTM could be enhanced by treating animals with the PKC activator bryostatin [36]. That is, exposing snails to bryostatin 24 h before snails were trained in hypoxic pond water with a single 0.5 h training session resulted in an LTM that persisted for at least 96 h. As we have shown here snails trained in hypoxic pond water with a 0.5 h training session are only able to form a 3 h memory (i.e. ITM). Thus, bryostatin which is a PKC agonist enhances a snails ability to produce LTM. Byrostatin's ability to enhance LTM formation was dependent on the soma, and thus the genes of RPeD1 to be intact, indicating again that molecular cascades leading to altered gene activity in this neuron are necessary for LTM formation. More recently, through a series of proteomic experiments, we observed that a specific PKC isoform (PKC epsilon) was found to be present only in the nervous tissue of animals that had been trained to form a LTM and not controls [37]. Future experiments will have to be performed to block these individual isoforms of PKC more precisely, to determine if the action of one distinct form is required for ITM or LTM. These experiments would also serve to reinforce the present data, as GF109203X has also been observed to inhibit the glycogen synthase kinase 3 (GSK-3) in addition to PKC [54]. Thus the use of an isoform selective inhibitor would also help rule out the possibility that GSK-3 inhibition affected the formation of memory in the present experiments.

The MAPK's are another family of kinases that regulate a diverse number of cellular processes [55]. MAPK activity has been shown to be necessary for the formation of memory in a number of different animals and training paradigms. In *Aplysia *MAPK was found to translocate to the nucleus following the presentation of stimuli that result in long-term facilitation at the sensory to motor neuron synapse [56]. In rats both cued and contextual fear conditioning were found to result in the activation of MAPK in the hippocampus, and inhibiting MAPK prevented memory formation [57]. Previously, in *Lymnaea *it was shown that single-trial food-reward appetitive classical conditioning training induced a rise in MAPK phosphorylation [41]. Moreover, these same authors showed that memory formation following this form of classical conditioning could be abolished by treating animals with a MAPK inhibitor [41]. Here we extend these findings using an operant conditioning training procedure and show that inhibiting MAPK activity blocks the formation of both ITM and LTM without altering basal aerial respiratory behaviour. Work from the Tonegawa lab [58] provides evidence of MAPK as a key regulator of translation during learning, as it's inhibition resulted in the block of translation factors eIF4E, 4EBP1 and ribosomal S6 phosphorylation. It is perhaps via these translational activators that MAPK works during normal memory formation, specifically during ITM where only translation is required. If this is indeed how MAPK works to lead to the formation of LTM then it would provide correlative evidence that for LTM to form ITM must first form [59,60]. That is, the molecular processes underlying LTM formation build on the molecular processes that cause ITM.

Learning and the production of a subsequent memory are critical to animal survival. As would be expected with such important processes, learning and memory are observable across a vast array of species from the simple worm *C. elegans *to humans. It is thus reasonable to hypothesize that such a fundamentally conserved mechanism of survival and adaptation, may occur as the result of a well conserved set of underlying molecular mechanisms. Here we provide some evidence of this by showing that memory formation in the pond snail Lymnaea relies on a set of core molecules (NMDA, PKC, MAPK) that have also been seen to be required in a number of other animals. From this base knowledge of the molecular workings of *Lymnaea *memory, we can now continue to elucidate the mechanisms involved in memory formation in order to fully understand how it can both occur, and be regulated either positively or negatively.

## Conclusions

In this study we showed that blockade of NMDA receptors, inhibition of MAPK and PKC activity prevent the formation of both ITM and LTM.

## Competing interests

The authors declare that they have no competing interests.

## Authors' contributions

DR and KL designed the experiments; DR performed the majority of the experiments; KL wrote the final version of paper. All authors read and approved the final manuscript.
